# Supplementary material for: Eoarchean and Hadean melts reveal arc-like trace element and isotopic signatures
Source: Nat Commun. 2023 Feb 28;14:1140. doi: 10.1038/s41467-023-36538-5 (PMC9975215; doi:10.1038/s41467-023-36538-5)
Supplement: Supplementary file 1 — Supplementary information [file 41467_2023_36538_MOESM1_ESM.pdf]

Supplementary Information for

Eoarchean and Hadean melts reveal arc-like trace element and isotopic signatures

Wriju Chowdhury[1*], Dustin Trail[1], Martha Miller[1], Paul Savage[2]

[1] Department of Earth & Environmental Sciences, University of Rochester, Rochester, NY 14627, USA.

[2] School of Earth and Environmental Sciences, University of St Andrews, Bute Building, Scotland KY16 9TS, UK

*Corresponding author: Wriju Chowdhury (wchowdhu@ur.rochester.edu)

This document includes

Supplementary Figs. 1-8

Si/O isotopic composition of Jack Hills zircons (JHZs)

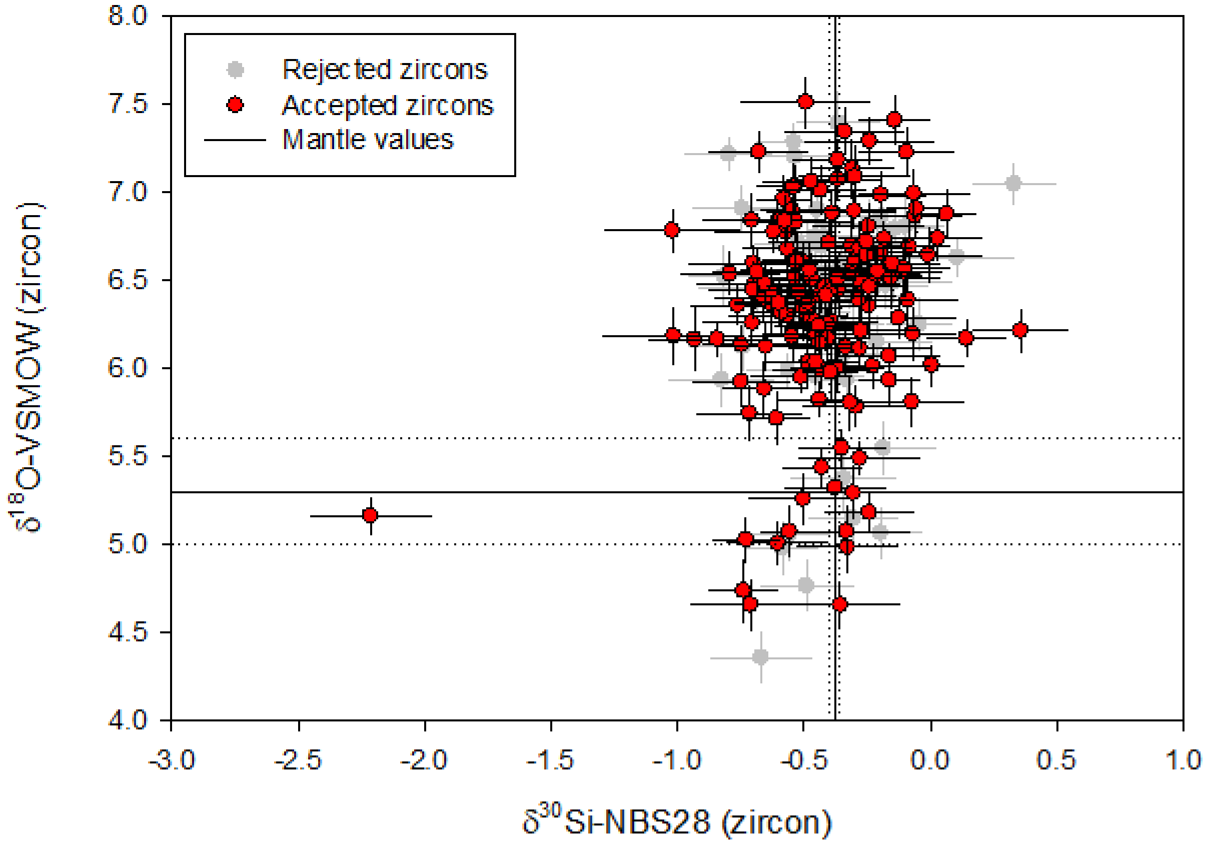

Supplementary Fig. 1: **δ^{30}Si and δ^{18}O values (±2 s.e.) of Hadean and Archean zircons discussed in this study** (Supplementary Table 1). Note the two sub-populations in this figure. Some analyses were rejected if a grain crack was noticed in the sputtering pit. The black lines are mantle zircon values. Mantle δ^{30}Si and δ^{18}O values from [1]. The δ^{30}Si mantle zircon value is an average of mantle-derived zircon megacrysts[20].

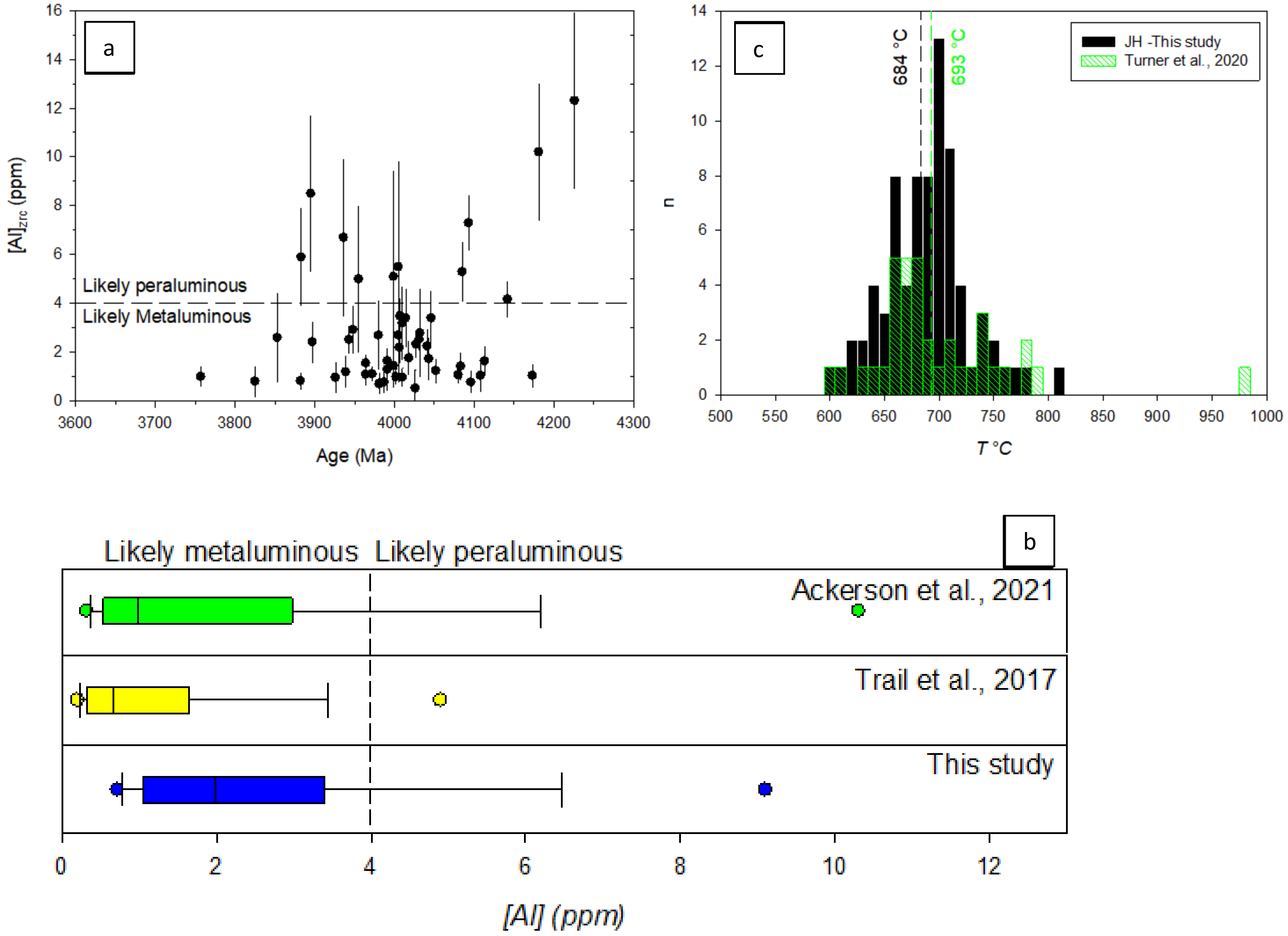

Supplementary Fig. 2 Aluminosity and Crystallization T of Jack Hills zircons. **a**. [Al] (± 2 s.e.) content of our JHZs (Jack Hills Zircons) plotted vs age. The aluminosity for JHZs is unchanged from the Hadean to the Archean. **b.** [Al] content of JHZs from this study compared to those reported in [2] and [3]. This study, as with the other two, shows ~83% of zircons that indicate metaluminous melts. The data points in each panel are the 5th and 95Th percentile of each dataset. **c.** Crystallization T of our JHZs compared to those of [4]. All temperatures have been derived using the Ti-in-zircon thermometer[5].

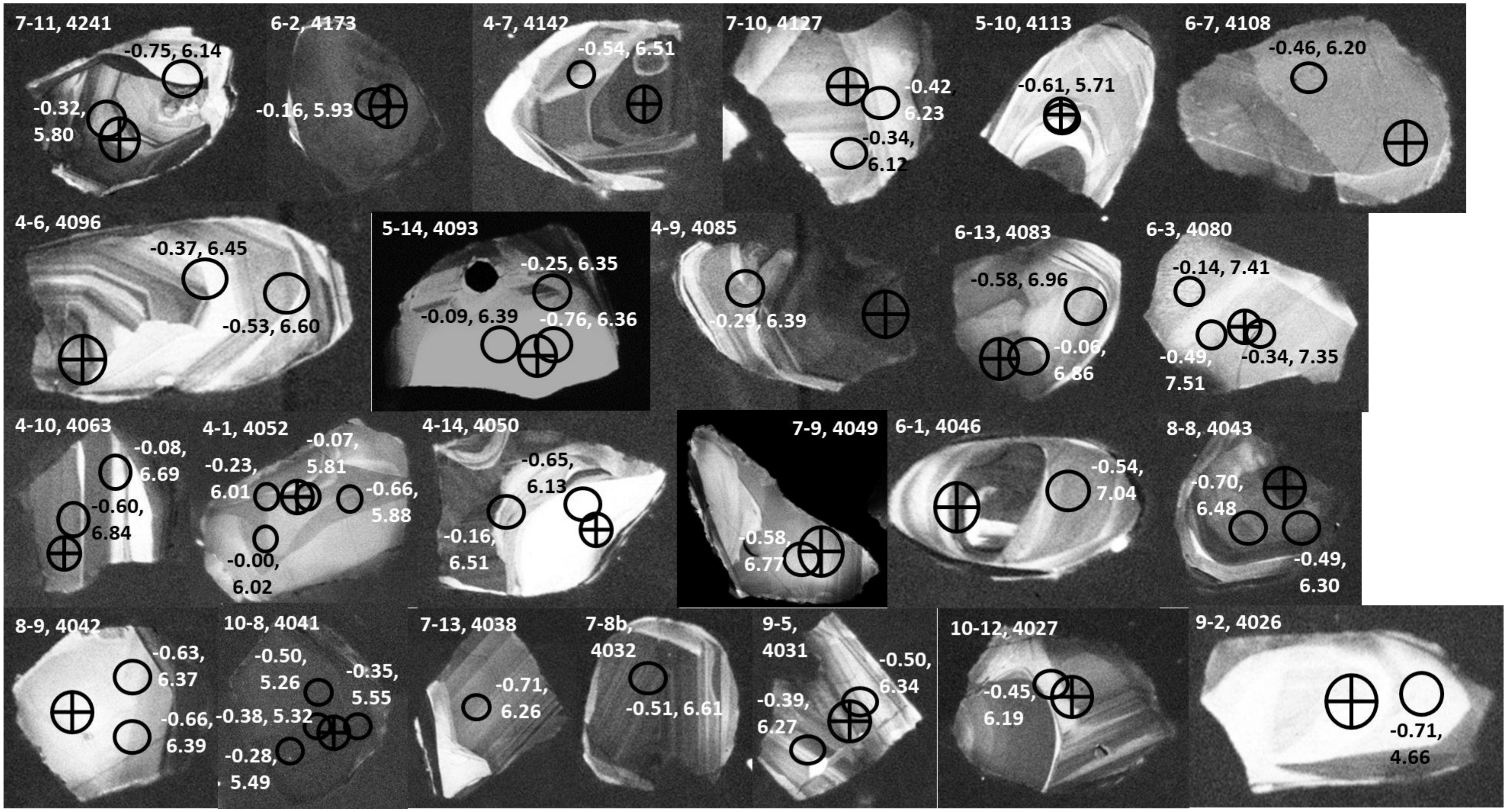

7-11, 4241
-0.75, 6.14
-0.32, 5.80
6-2, 4173
-0.16, 5.93
4-7, 4142
-0.54, 6.51
7-10, 4127
-0.42, 6.23
-0.34, 6.12
5-10, 4113
-0.61, 5.71
6-7, 4108
-0.46, 6.20
4-6, 4096
-0.37, 6.45
-0.53, 6.60
5-14, 4093
-0.25, 6.35
-0.09, 6.39
-0.76, 6.36
4-9, 4085
-0.29, 6.39
6-13, 4083
-0.58, 6.96
-0.06, 6.86
6-3, 4080
-0.14, 7.41
-0.49, 7.51
-0.34, 7.35
4-10, 4063
-0.08, 6.69
-0.60, 6.84
4-1, 4052
-0.23, 6.01
-0.07, 5.81
-0.66, 5.88
-0.00, 6.02
4-14, 4050
-0.65, 6.13
-0.16, 6.51
7-9, 4049
-0.58 6.77
6-1, 4046
-0.54, 7.04
8-8, 4043
-0.70, 6.48
-0.49, 6.30
8-9, 4042
-0.63, 6.37
-0.66, 6.39
10-8, 4041
-0.50, 5.26
-0.35, 5.55
-0.38, 5.32
-0.28, 5.49
7-13, 4038
-0.71, 6.26
7-8b, 4032
-0.51, 6.61
9-5, 4031
-0.50, 6.34
-0.39, 6.27
10-12, 4027
-0.45, 6.19
9-2, 4026
-0.71, 4.66

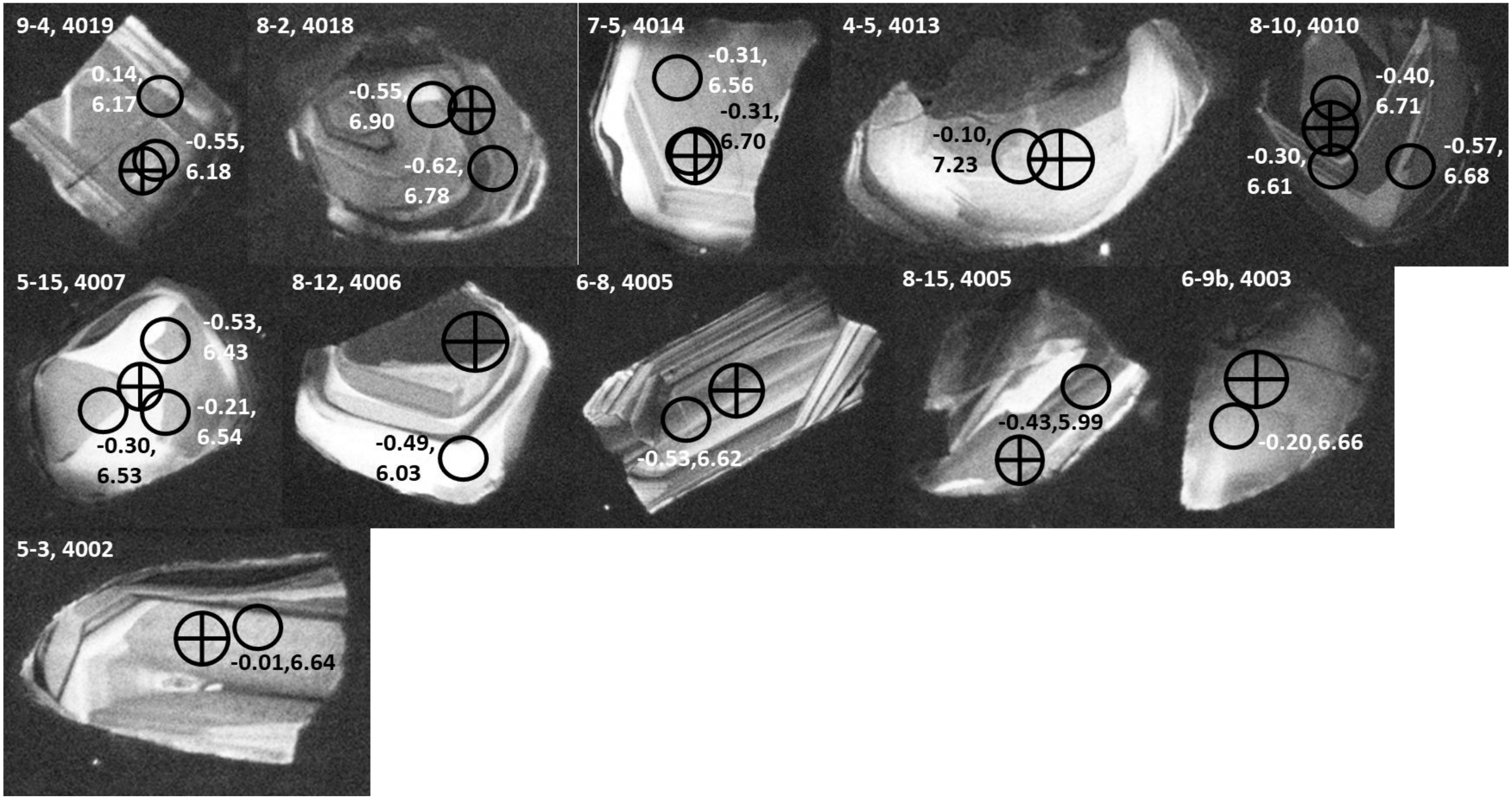
9-4, 4019
0.14,
6.17
-0.55,
6.18
8-2, 4018
-0.55,
6.90
-0.62,
6.78
7-5, 4014
-0.31,
6.56
-0.31,
6.70
4-5, 4013
-0.10,
7.23
8-10, 4010
-0.40,
6.71
-0.30,
6.61
-0.57,
6.68
5-15, 4007
-0.53,
6.43
-0.21,
6.54
-0.30,
6.53
8-12, 4006
-0.49,
6.03
6-8, 4005
-0.53,6.62
8-15, 4005
-0.43,5.99
6-9b, 4003
-0.20,6.66
5-3, 4002
-0.01,6.64

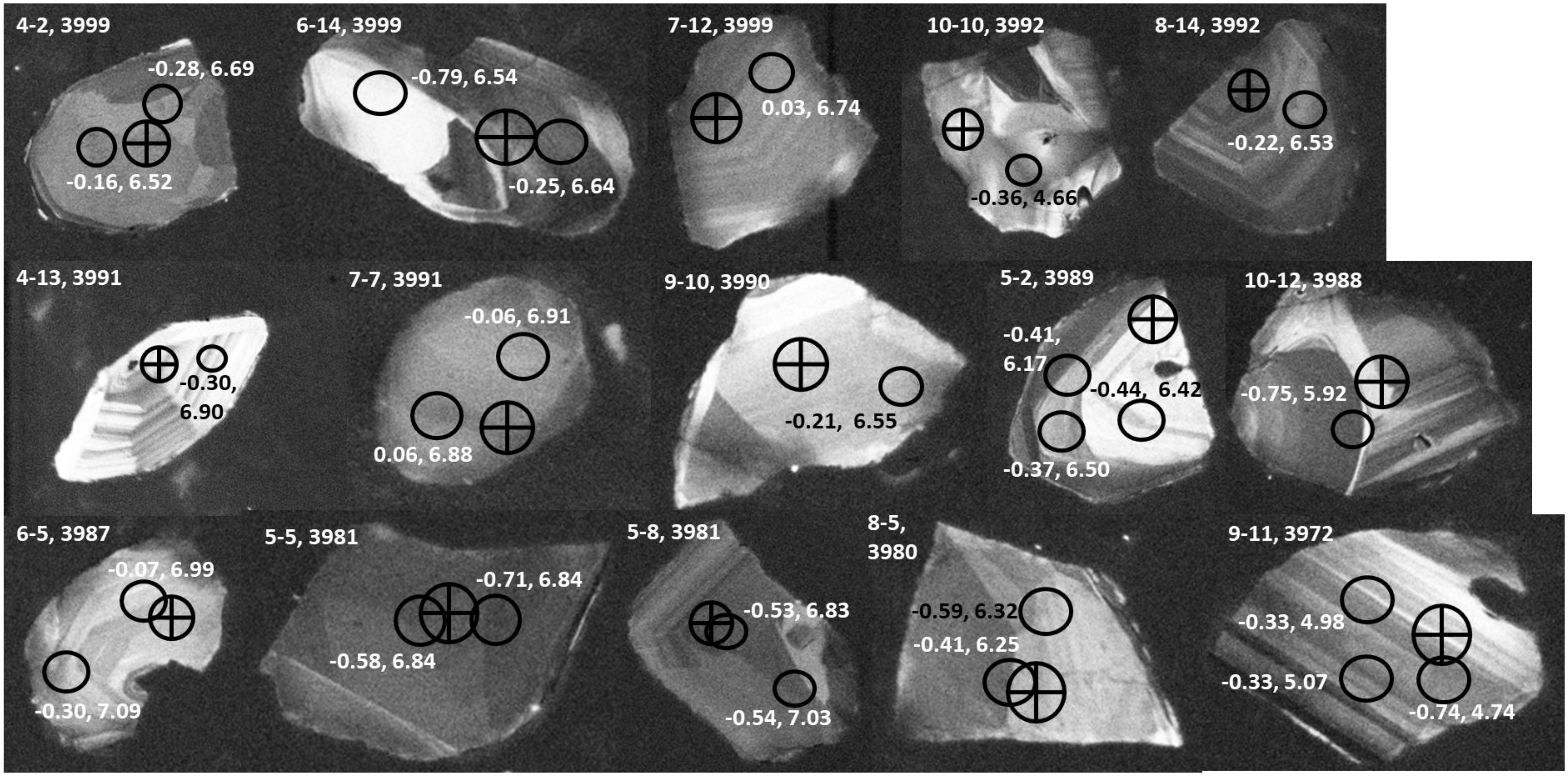
4-2, 3999
-0.28, 6.69
-0.16, 6.52
6-14, 3999
-0.79, 6.54
-0.25, 6.64
7-12, 3999
0.03, 6.74
10-10, 3992
-0.36, 4.66
8-14, 3992
-0.22, 6.53
4-13, 3991
-0.30, 6.90
7-7, 3991
-0.06, 6.91
0.06, 6.88
9-10, 3990
-0.21, 6.55
5-2, 3989
-0.41, 6.17
-0.44, 6.42
-0.37, 6.50
10-12, 3988
-0.75, 5.92
6-5, 3987
-0.07, 6.99
-0.30, 7.09
5-5, 3981
-0.71, 6.84
-0.58, 6.84
5-8, 3981
-0.53, 6.83
-0.54, 7.03
8-5, 3980
-0.59, 6.32
-0.41, 6.25
9-11, 3972
-0.33, 4.98
-0.33, 5.07
-0.74, 4.74

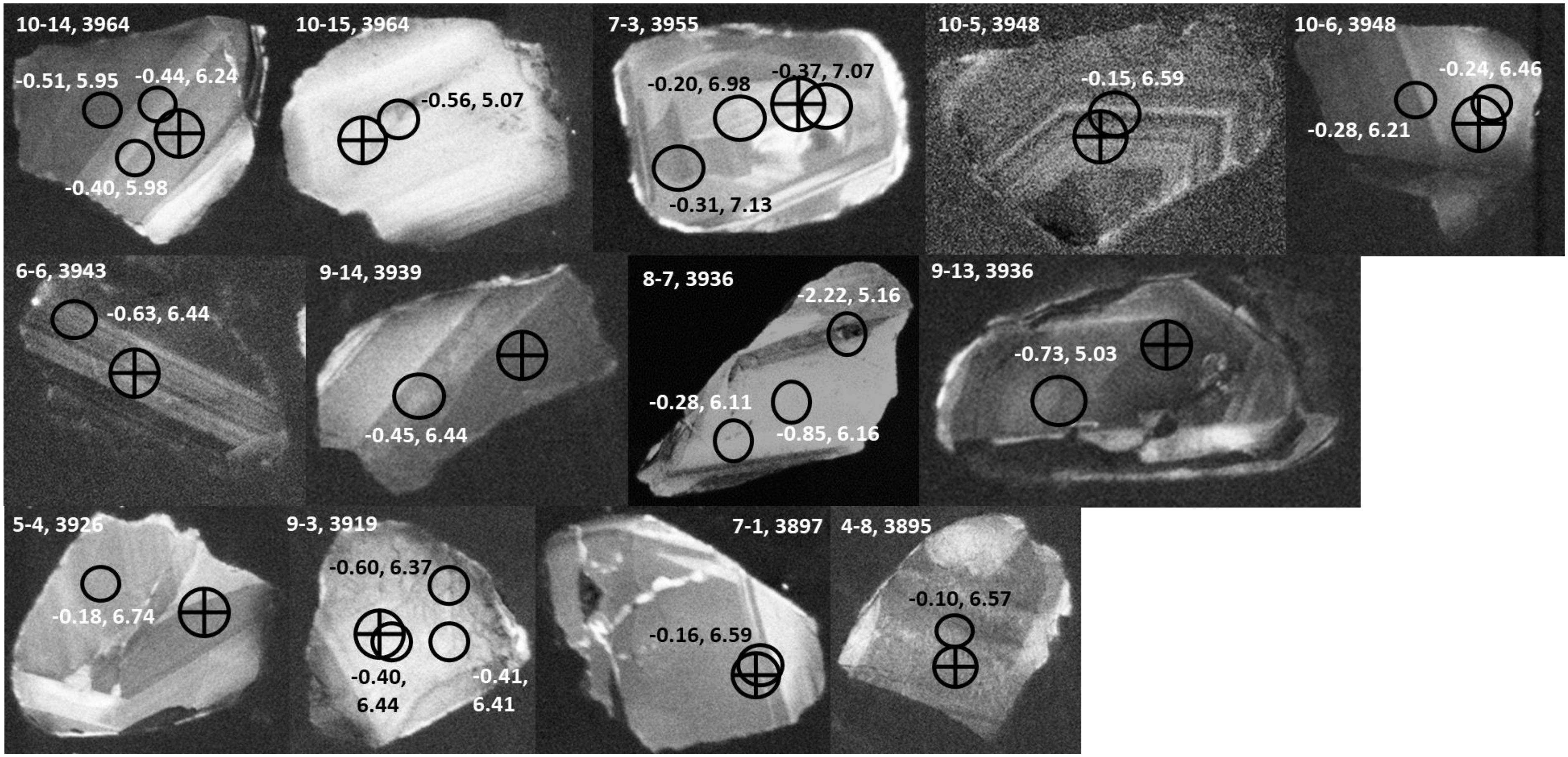
10-14, 3964
-0.51, 5.95
-0.44, 6.24
-0.40, 5.98
10-15, 3964
-0.56, 5.07
7-3, 3955
-0.20, 6.98
-0.37, 7.07
-0.31, 7.13
10-5, 3948
-0.15, 6.59
10-6, 3948
-0.24, 6.46
-0.28, 6.21
6-6, 3943
-0.63, 6.44
9-14, 3939
-0.45, 6.44
8-7, 3936
-2.22, 5.16
-0.28, 6.11
-0.85, 6.16
9-13, 3936
-0.73, 5.03
5-4, 3926
-0.18, 6.74
9-3, 3919
-0.60, 6.37
-0.40, 6.44
-0.41, 6.41
7-1, 3897
-0.16, 6.59
4-8, 3895
-0.10, 6.57

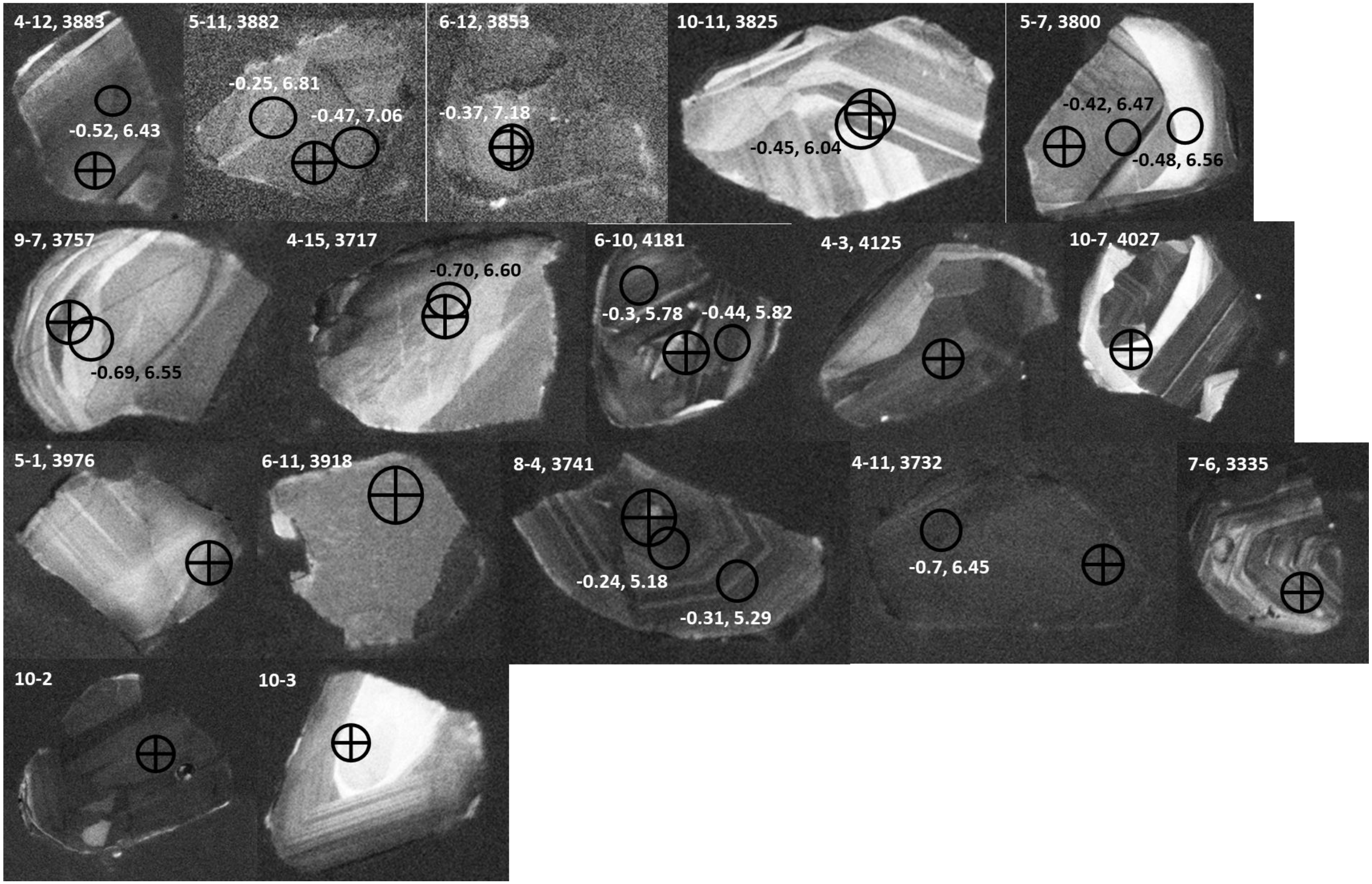
4-12, 3883
-0.52, 6.43
5-11, 3882
-0.25, 6.81
-0.47, 7.06
6-12, 3853
-0.37, 7.18
10-11, 3825
-0.45, 6.04
5-7, 3800
-0.42, 6.47
-0.48, 6.56
9-7, 3757
-0.69, 6.55
4-15, 3717
-0.70, 6.60
6-10, 4181
-0.3, 5.78
-0.44, 5.82
4-3, 4125
10-7, 4027
5-1, 3976
6-11, 3918
8-4, 3741
-0.24, 5.18
-0.31, 5.29
4-11, 3732
-0.7, 6.45
7-6, 3335
10-2
10-3

Supplementary Fig. 3. CL (Cathodoluminiscence) images of the JHZs (Jack Hills Zircon) discussed in this study. The LA (Laser Ablation) spots for geochronology were placed directly on top of the SIMS (Secondary Ion Mass Spectrometer) analysis locations. LA spots shown here are locations for TE (Trace Element) measurements.

Legend:

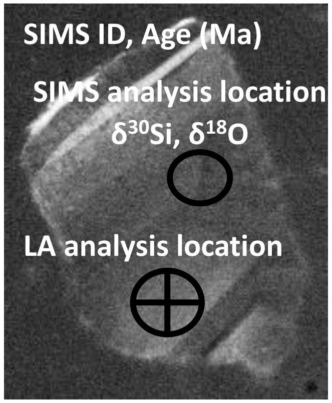

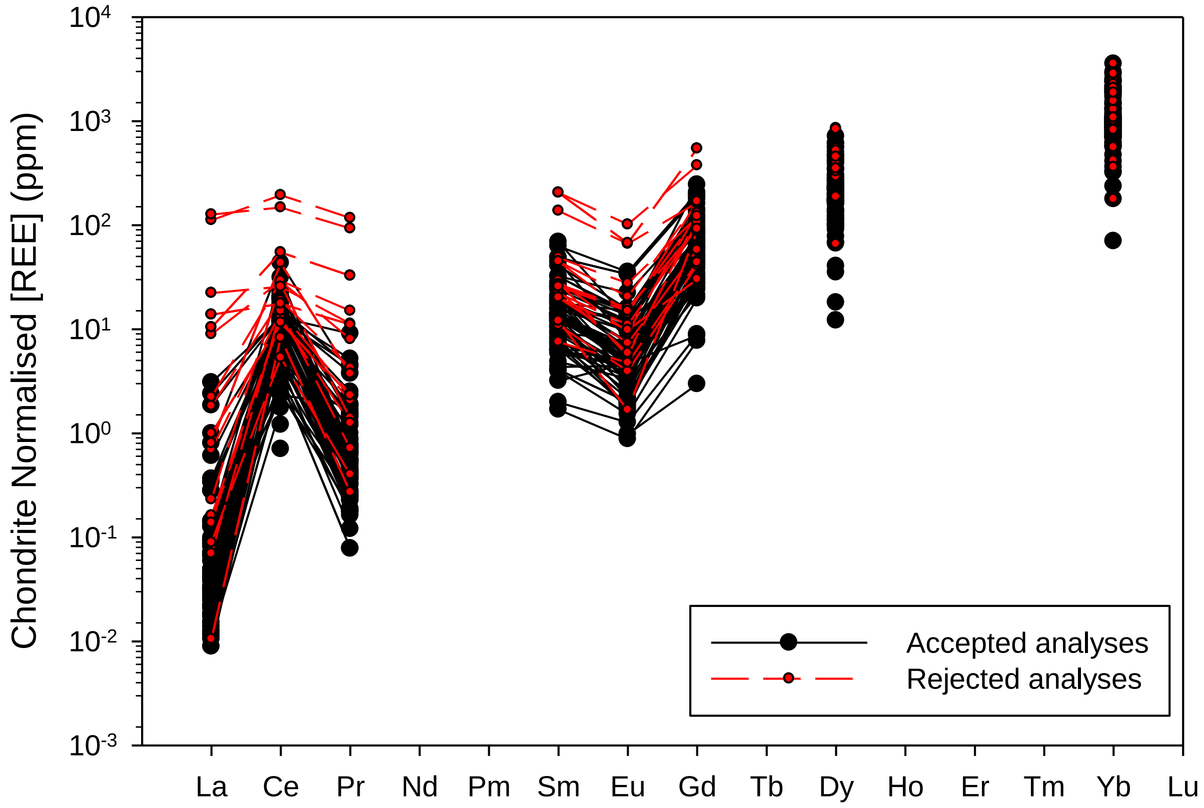

Supplementary Fig. 4: REE (Rare earth element) patterns of JHZs (Jack Hills Zircon) we analyzed. Our zircons showing characteristic low LREE (Light-REE), high HREE (Heavy-REE), positive Ce anomalies and negative Eu anomalies. (See Methods for rejection criteria). The gaps in the pattern are because all not all REEs were analyzed.

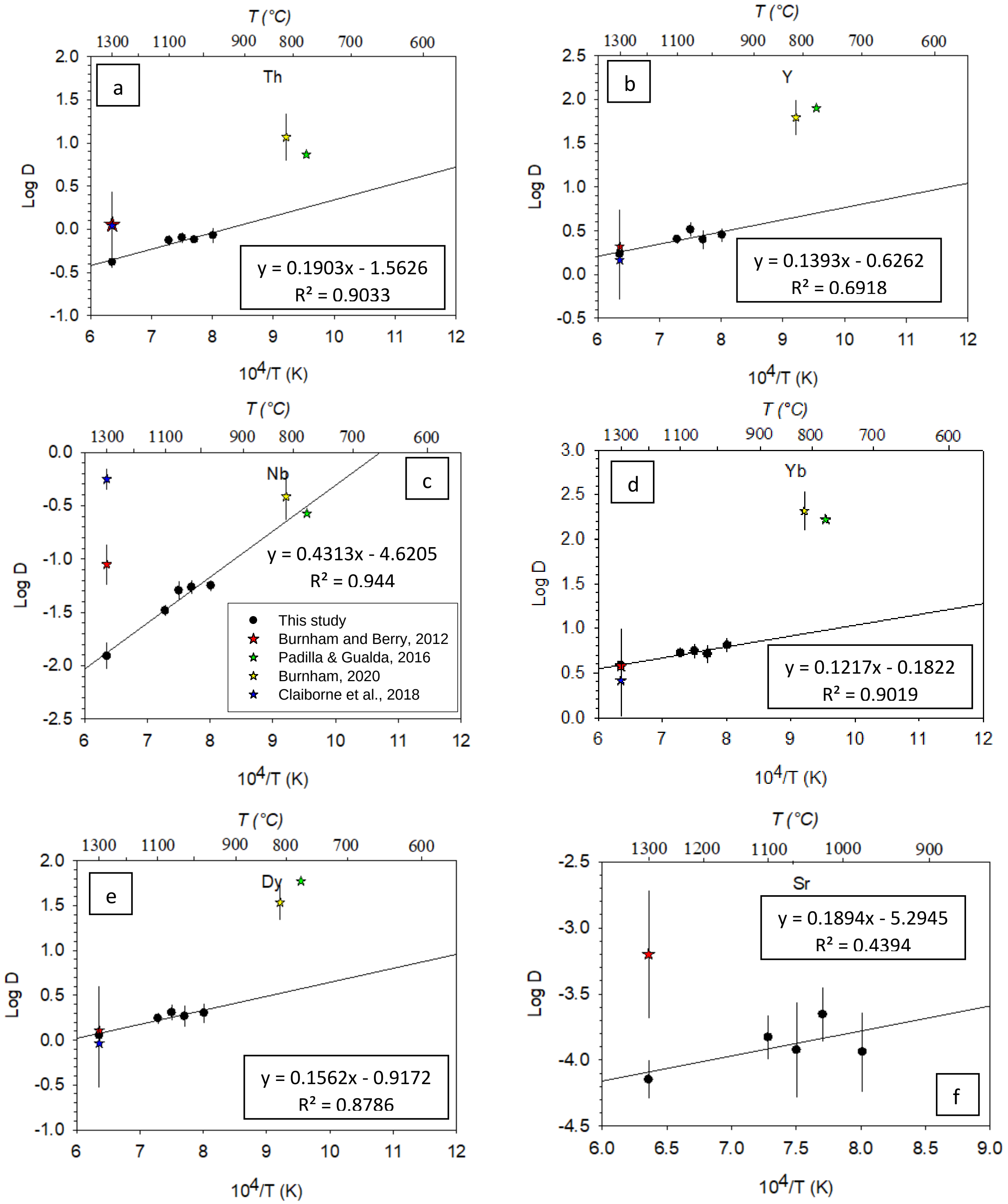

Supplementary Fig. 5 (a-f): D (Partition coefficient) (±2 s.e.) values measured in this study compared to previous studies[6-9]. The regressions shown here are only through our experimental D-values ($[X]_{zrc}/[X]_{melt}$ where [X] is the concentration of elements of interest) which are represented by solid circles. These regressions are used to derive the JHZ (Jack Hills Zircon) melt TE (Trace Element) values in Fig. 4.

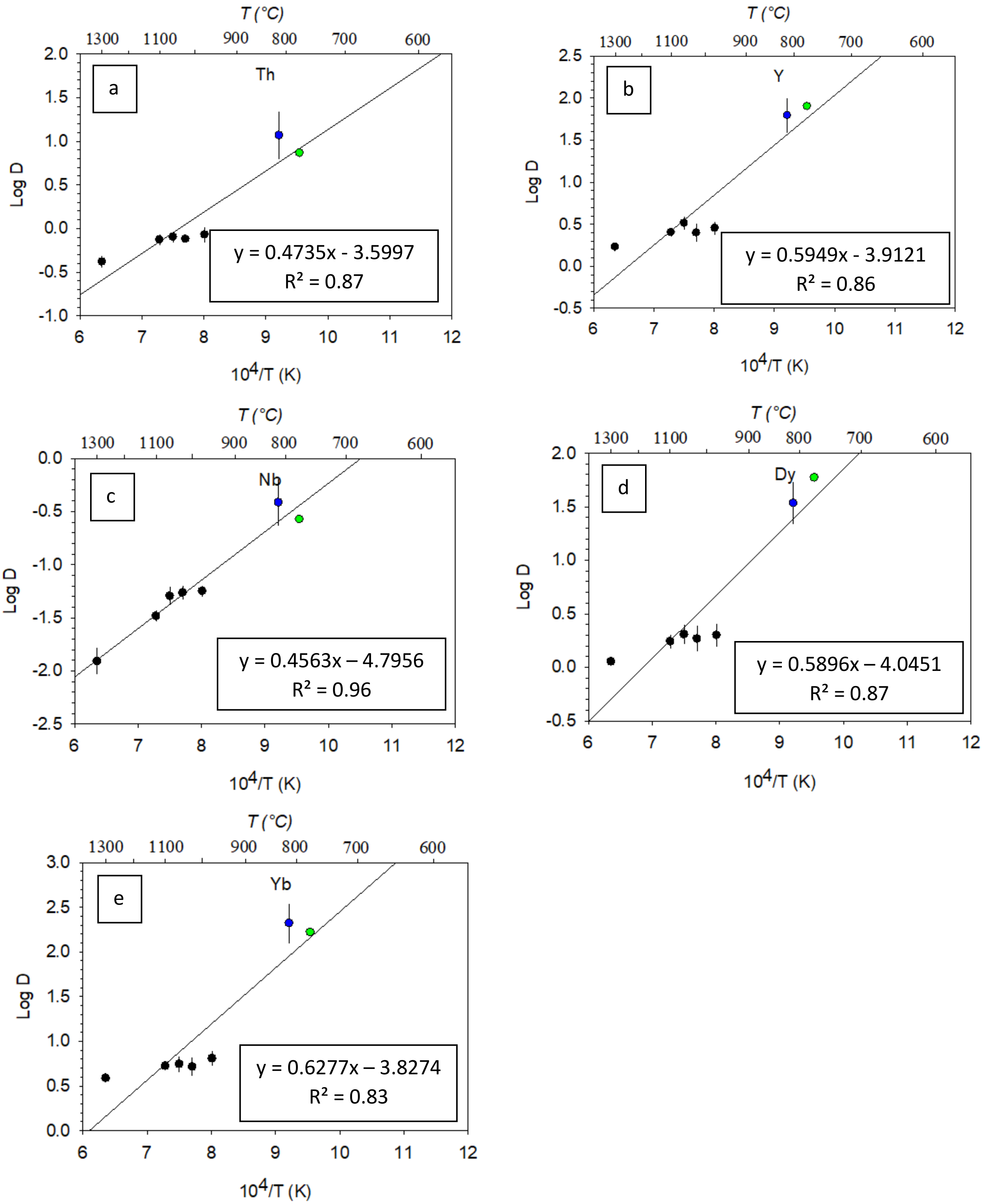

Supplementary Fig. 6 (a-e): Regressions obtained after combining the experimental data from this study and the natural data. Natural data from [7] (Green circles; T = 775 °C) and [9] (blue circles; T = 813 °C). Error bars are 2 s.e.

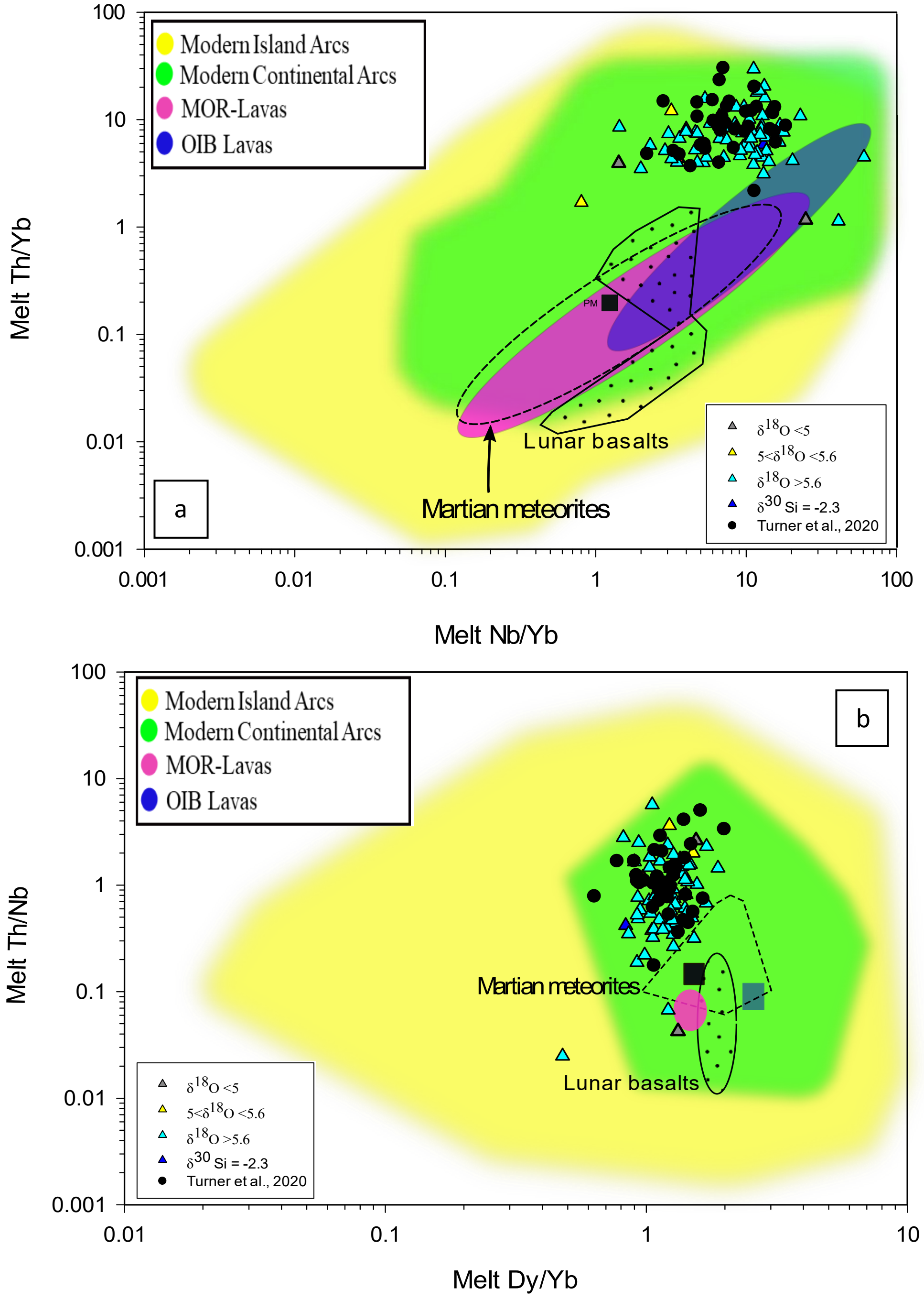

Supplementary Fig. 7 (a and b): **Discrimination diagrams obtained from D (Partition Coefficient) values using the regressions of Supplementary Fig. 6**. These diagrams are unlike Fig 4 which are derived using the regressions of Supplementary Fig. 5. Note that independent of the regression used, it does not change the overall interpretation of our results. However, we prefer to use Supplementary Fig. 5, because the experimental zircons crystallize in a more controlled and defined setting than the partition coefficients presented from natural studies[7, 9].

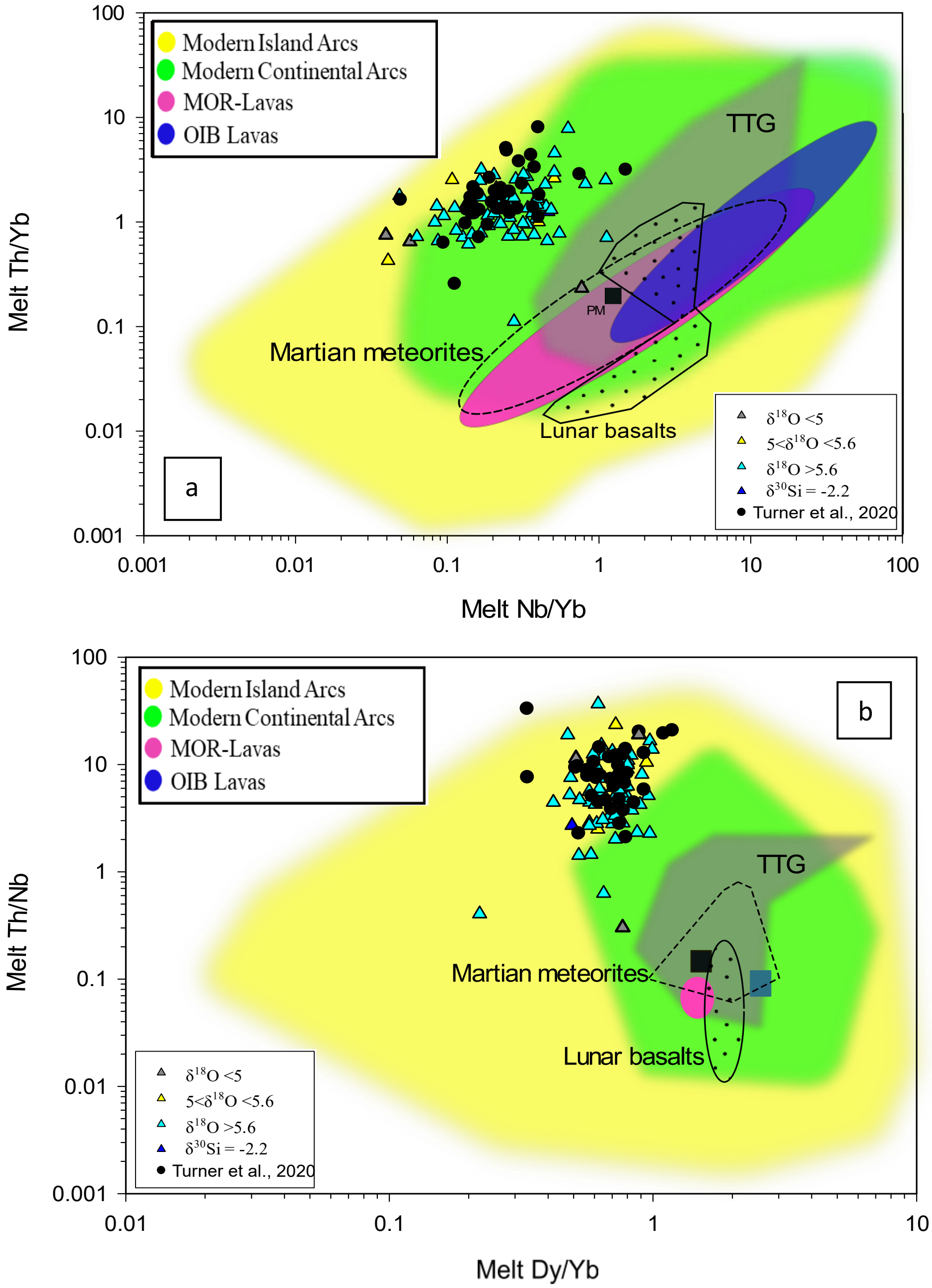

Supplementary Fig. 8 (a and b): Figure 4 modified by adding a TTG (Tonalite-Trondjhemite-Granodiorite) field (Data from GEOROC database). This figure shows that our JHZ (Jack Hills Zircon) model melts have different chemical signatures than TTGs.

SUPPLEMENTARY REFERENCES

1. Trail, D., P. Boehnke, P. S. Savage, M.-C. Liu, M. L. Miller & I. Bindeman. Origin and significance of Si and O isotope heterogeneities in Phanerozoic, Archean, and Hadean zircon. Proc. Natl. Acad. Sci.115: 10287-10292 (2018).
2. Ackerson, M.R., Trail, D. and Buettner, J. Emergence of peraluminous crustal magmas and implications for the early Earth. *Geochem. Perspect. Lett.*, **17**, 50-54, (2021).
3. Trail, D., Tailby, N., Wang, Y., Mark Harrison, T., and Boehnke, P. Aluminum in zircon as evidence for peraluminous and metaluminous melts from the Hadean to present, *Geochem. Geophys. Geosyst.*, **18**, 1580– 1593, doi:10.1002/2016GC006794 (2017).
4. Turner, S., Wilde, S., Wörner, G., Schaefer, B. & Lai, Y.-J. An andesitic source for Jack Hills zircon supports onset of plate tectonics in the Hadean. *Nat. Commun.* **11**, 1241-1245, (2020).
5. Ferry, J. M. & Watson, E. B. New thermodynamic models and revised calibrations for the Ti-in-zircon and Zr-in-rutile thermometers. *Contrib. Mineral. Petr.* **154**, 429-437 (2007).
6. Burnham, A. D. & Berry, A. J. An experimental study of trace element partitioning between zircon and melt as a function of oxygen fugacity. *Geochim. Cosmochim. Ac.* **95**, 196-212, (2012)
7. Padilla, A. J. & Gualda, G. A. R. Crystal-melt elemental partitioning in silicic magmatic systems: An example from the Peach Spring Tuff high-silica rhyolite, Southwest USA. *Chem. Geol.* **440**, 326-344 (2016).
8. Burnham, A.D. Key concepts in interpreting the concentrations of the rare earth elements in zircon. *Chem. Geol.* **551,** 119765 (2020).
9. Claiborne, L. L., Miller, C. F., Gualda, G. A., Carley, T. L., Covey, A. K., Wooden, J. L., & Fleming, M. A. Zircon as magma monitor: Robust, temperature-dependent partition coefficients from glass and zircon surface and rim measurements from natural systems. *Microstructural geochronology: Planetary records down to atom scale*, 1-33. (2018).
