# Supplementary material for: Eoarchean and Hadean melts reveal arc-like trace element and isotopic signatures
Source: Nat Commun. 2023 Feb 28;14:1140. doi: 10.1038/s41467-023-36538-5 (PMC9975215; doi:10.1038/s41467-023-36538-5)
Supplement: Supplementary file 3 — Description of Additional Supplementary Files [file 41467_2023_36538_MOESM3_ESM.pdf]

Description of Additional Supplementary Files:

Supplementary Data 1: Partition Coefficients experimentally derived for this study and those previously published.

Supplementary Data 2: LA-ICP-MS measurements of Jack Hills Zircons and Kuehl Lake standard.

Supplementary Data 3: SIMS measurements of Jack Hills Zircons and derived delta values

Supplementary Data 4: Derived delta values of Jack Hills model parent melts and ODP serpentinites

Supplementary Data 5: Experimental details and LA-ICPMS measurements of experimental glass and zircons

Supplementary Data 6: Raw geochronology data of zircons measured before Si+O isotopic measurements

Supplementary Data 7: Raw geochronology data of zircons measured after Si+O isotopic measurements and reported in this study
